# HIV Treatment as Prevention: Contradictory Perspectives from Dynamic Mathematical Models

**DOI:** 10.1155/2014/760734

**Published:** 2014-12-15

**Authors:** Jing Wu, Jessie L. Norris, Yujiang Jia, Ning Wang

**Affiliations:** ^1^National Center for AIDS/STD Control and Prevention, Chinese Center for Disease Control and Prevention, No. 155 Changbai Road, Beijing 102206, China; ^2^School of Agroforestry and Medicine, The Open University of China, Beijing 100039, China; ^3^Department of Preventive Medicine, School of Medicine, Vanderbilt University, Nashville, TN 37232, USA

## Abstract

The preventative effects of antiretroviral therapy for people with HIV have been debated since they were first raised. Models commenced studying the preventive effects of treatment in the 1990s, prior to initial public reports. However, the outcomes of the preventive effects of antiretroviral use were not consistent. Some outcomes of dynamic models were based on unfeasible assumptions, such as no consideration of drug resistance, behavior disinhibition, or economic inputs in poor countries, and unrealistic input variables, for example, overstated initiation time, adherence, coverage, and efficacy of treatment. This paper reviewed dynamic mathematical models to ascertain the complex effects of ART on HIV transmission. This review discusses more conservative inputs and outcomes relative to antiretroviral use in HIV infections in dynamic mathematical models. ART alone cannot eliminate HIV transmission.

## 1. Introduction

Dynamic mathematical models have been applied to examine the impact of antiretroviral therapy (ART) on HIV transmission since the early 1990s [1]. An early model [1] demonstrated that treatment of HIV-infected individuals promoted the spread of the virus, due to an unrealistic assumption that ignored the virus's reduced infectiousness through ART. Blower et al. explored the effect of ART on new HIV infections and the transmission of resistance in different scenarios in San Francisco gay communities [2–4], but a potentially different conclusion was also reached in similar models in resource-constrained countries [5]. The predictions in the San Francisco studies were tested with “real-world” empirical data [6], and they both showed that the prevalence of transmitted drug resistance increased from 1996 to 2001 in San Francisco. In 2002, UNAIDS released a report that for the first time publicly acknowledged the preventive role of treatment for people with HIV [7]. Over the next 10 years many studies showed the potential impact of ART on new HIV infections based on various treatment-related scenarios and settings, but thus far the conclusions regarding HIV treatment for prevention have been inconsistent.

Timely diagnosis and retention in HIV treatment create a virtuous cycle promoted by the display of better quality of life and prolonged survival time for people with HIV. ART among people with HIV produces preventive benefits by decreasing the HIV plasma concentration in a given population [8–10] and reducing risk behavior through knowledge of infection status [11]. On the other hand, the use of ART may also facilitate HIV transmission due to a greater number of cases in the prevalence pool [12], behavior disinhibition [13], increased drug resistance [6], poor access to care services [14], and variations in the transmission routes of the targeted population [14]. Therefore, the paradoxes should be considered regarding the impacts of treatment as prevention, including both the preventive benefits and potential negative effects. It is thus critical to examine the relationship between HIV transmission and treatment from multidimensional perspectives.

Observational or experimental studies [10, 15] and a meta-analysis [9] demonstrated that the effectiveness of ART on HIV transmission prevention ranged widely from 50% to 96%, but the explicit effects need to be evaluated by continuous studies. Observational studies and clinical trials have limited the ability to demonstrate the long-term prevention effects of ART based on the dynamics of variables such as risk behaviors and drug resistance and the initiation and uptake of ART, as well as the roles of these variables in different settings. Dynamic mathematical models have provided an opportunity to examine the past, present, and future of HIV epidemics when the key parameters are available.

A series of studies discussed the use of HIV treatment as prevention and also debated existing paradoxes, as well as offering future directions on this topic [16, 17]. The WHO has special concerns for treatment as prevention and further assesses its effects under future evidence [18]. This paper reviewed the use of dynamic mathematical models in examining the complex effects of ART on HIV transmission.

## 2. Outcome Indicators to Evaluate HIV Transmission in Dynamic Models

Three key parameters to estimate HIV transmission in dynamic mathematical models are new HIV cases averted compared to a base scenario, incidence, and reproductive rate over time. HIV incidence and reproductive rate both consider the number of HIV-susceptible individuals and new HIV cases over a certain time period [19, 20], but the number of new HIV cases prevented is still a practical method for assessing the prevention effect of ART, which was applied in many dynamic models.

Changes in the above parameters in different scenarios over time may be observed if a specific or comprehensive intervention has preventive effects. The values of the incidence and reproductive rates over time will be the key outcome indicators to determine whether the HIV epidemic could be contained, eliminated, or eradicated [20, 21].

## 3. The Paradox of Treatment as Prevention in HIV-Infected Individuals: More HIV Infections versus Fewer New HIV Infections

The purpose of treatment is to save lives, as ART plays a therapeutic role after diagnosis. Compared to people not on treatment, those with HIV who are on treatment have greater longevity, lower mortality [22], and better quality of life, which increases the number of HIV survival cases in the pool of HIV-infected individuals. Studies [8, 23] have shown that those HIV-infected individuals receiving ART exhibit decreased plasma concentration of HIV, thereby decreasing the probability of HIV transmission. Thus there is a paradox between the increased number of cumulative HIV survivors and the number of new HIV cases, both of which are due to effective treatment. The result is that a balance is reached between HIV treatment effectiveness and HIV prevalence, which has been verified by Wilson [14].

## 4. Direct Input Values of Art in Dynamic Mathematical Models

Preventive effectiveness of ART alters with changes in treatment-related parameters such as initiation time, adherence, coverage, and efficacy of treatment. The time from infection to diagnosis is an important parameter in averting new HIV infections, which was indicated by comparison at different CD4 cell count levels [15]. Studies [21, 24–28] showed that there were fewer new HIV cases if treatment took place at higher levels of CD4 cell counts, while controlling for the other input values. ART can be initiated at different CD4 counts, from 200 cells/mm^3^ to 900 cells/mm^3^, or after HIV confirmation of diagnosis irrespective of CD4 counts. Controversies arise from the fact that the parameter is vague concerning the 2 to 38 times increase in HIV transmissibility during early HIV infection, compared to chronic HIV infection [26, 29–33] (see the supporting information on the website for references [29, 31]). The higher the relative infectiousness due to a high viral load of viremia in the early phase of HIV infection, the higher the proportion of new infections transmitted. The estimated proportion of new infections attributed to early phase infection was 0.4–38%, with a relative infectiveness of the above [26, 29–33]. AIDS Experts expressed divergent viewpoints about the effects of ART [16]. Relative infectiousness, duration of the early phase of infection, amount of HIV transmission attributed to viral load, and acceptability of referred articles were argued, so further evidence is needed. HIV transmission during the early infection phase is critical because it provides an indicator to resource-limited countries on how to allocate limited treatment resources to target populations in order to maximize the benefits at population level. Perhaps as a compromise it may be possible in the future to pool the above proportion of new infections that are due to early infection. An increased CD4 threshold for treatment eligibility produces economic burdens in resource-constrained countries [34]. However, treatment eligibility that is economically inaccessible in limited-resource countries will not produce real benefits or result in subsequent cost reductions [31, 34].

Treatment coverage can have two descriptions, depending on the denominators: among eligible HIV infections, or among all HIV infections. The former is a concept often discussed relative to treatment initiation time. Clearly, ART plays a role in controlling HIV transmission compared to the counterfactual scenario without ART [35, 36], and other studies [24, 27, 28, 37–39] have shown that the greater the coverage, the greater the number of HIV infections averted and the lower the number of new HIV infections, incidences, and reproductive rates. An example in Papua New Guinea emphasized the importance of treatment coverage [40]. If the numbers of ART decreased from 50% to 90% compared to the ones from 2009, incidence and AIDS-related deaths in the next five years would increase from 68 to 100% and from 70 to 100%, respectively. However, we should be concerned that low treatment coverage among HIV patients with CD4 ≤ 200/mm^3^ or patients whose HIV has already developed into AIDS may not produce preventive public health outcomes [13, 24, 25] but only therapeutic effects. Treatment efficacy is another parameter to be considered in the models for evaluating the effects of ART. Treatment efficacy is embodied by a decreasing viral load and subsequently decreased infectiousness among a target population. The 96% efficacy of ART to reduce HIV heterosexual transmission reported by the randomized-controlled HPTN 052 study [15] has been applied in recent modeling studies, but with severely unrealistic conditions, for example, treatment-naïve HIV infections and high adherence of ART [41]. In addition, more and more people experiencing treatment failure also reduce the efficacy of treatment over time. Some modelers considered treatment efficacy differences based on transmission type [42] and/or virological suppression level of HIV index on treatment [25, 43], or on different outcomes from different studies [13], and the disparate values entered into the models were among the parameters affecting the output outcomes. A negative relationship between ART efficacy and HIV transmission has already been established in several studies [3, 27, 35, 36, 42, 44]. Adherence is an important index to determine treatment efficacy. Higher adherence levels prevent more new HIV infections [26, 37], but screening for adherence may not have any impact on HIV transmission [45]. Ultimately, the above treatment-related parameters are combined and intensified to improve the number of new HIV infections prevented.

Many models [27, 28, 35, 46] considered the status quo of ART or WHO ART guidelines as the reference. However, some models proposed impractical assumptions, such as the assumption that all infections could be treated at a high CD4 count level or indeed at any level. First, not all infections can be identified because of HIV's long asymptomatic period. Second, some resource-limited countries may not have sufficient funding to treat “all” infections. Assuming a high or complete treatment coverage is not practical irrespective of whether it is among the high-risk population or populations at different CD4 counts or the total population [26, 27, 42–44]. The overly optimistic assumption of “all” infections cannot be even determined, let alone treated. Yet most researchers considered realistic coverage to be 50%, similar to that in the United States (about 40%) [47]. Treatment efficacy with high adherence is similar to the HPTN052 study [15], but in the real world ART effectiveness is lower and wanes over time due to behavior disinhibition, increases in live infections, reduced adherence, and subsequent resistance [14, 27]. The dynamics of treatment efficacy are almost entirely ignored in mathematical models.

In conclusion, ART use can reduce new HIV cases. However, treatment alone cannot eliminate HIV epidemics but rather contain the epidemic if considered in a realistic situation [25, 42, 48].

## 5. Drug Resistance as a Concomitant Factor of HIV Treatment as Prevention

Drug resistance is one concomitant factor of HIV treatment as prevention. Resistance is manifested from the mutation of the HIV virus with or without ART use. Data from the models and empirical data both testify to an increasing trend of resistance over time [6, 49, 50]. Comparatively, drug resistance due to preexposure prophylaxis (PrEP) was significantly lower than resistance due to ART use among people with HIV [27], so treatment-related parameters are the key factors in drug resistance [27]. However, drug resistance on PrEP cannot be neglected [51]. Transmitted drug-resistant viruses will have a great impact on HIV mortality in the future [49]; therefore, it is imperative to assiduously monitor the dynamics of drug resistance, especially in resource-limited countries [52].

A series of models by Abbas et al. [27, 53, 54] examined the relationship between PrEP and its consequent resistance. PrEP coverage, adherence, drug resistance development among new patients who were previously uninfected PrEP users, HIV testing frequency, and inadvertent PrEP use among the susceptible population are the key PrEP-related factors affecting drug-resistant prevalence [27, 54, 55]. Furthermore, factors such as PrEP coverage, persistence time of transmitted resistance, and negligent PrEP use among the uninfected [27, 54] determined the resistance transmission. HIV testing frequency, PrEP coverage, and the duration of negligent PrEP use among the susceptible population had an impact on acquired resistance among the previously uninfected [54]. However, inadvertent PrEP use in the primary stage of HIV had almost no effect on the prevalence of drug resistance but resulted in a significant increase in resistance levels in the chronic stage of HIV [27]. Surprisingly, if risk behaviors in a population are kept stable, PrEP will lead to a paradox: the proportion of new HIV infections due to resistant viruses will increase, but the number of new HIV infections due to resistant viruses will decrease [51]. PrEP use under stable risk behaviors decreases the transmission of both HIV and resistant HIV. In the future we should address the indefinite influences on the therapeutic and preventive efficacy of ART among infected individuals who were inadvertent PrEP users. It is an intractable resistance problem and indicates a need to find a balance for ART use between susceptible population and those already infected.

An aggressive treatment strategy could produce a high level of drug resistance, which could minimize or counterbalance the benefit of preventive effectiveness of ART [25, 50]. One example in southern Asia disclosed the potential severity of HIV resistance: in the absence of resistance monitoring or optimal treatment, the proportion of drug-resistant infections is expected to account for one-fourth of new HIV infections over the 10 years following expanded ART [56]. In addition, other factors also play a less important role in the prevalence of HIV resistance. The occurrence time of major resistant mutations after treatment rollout and the adaptive capacity of drug resistance are sensitive factors affecting drug resistance prevalence among treatment-naïve infections [56], but random mutation is the main contributing factor to drug resistance at treatment initiation [57]. Sensitive factors of drug resistance among treated cases include the recurrent time of drug resistance on treatment and the prevalence of acquired transmitted resistance [56]. Duration of transmitted resistance, transmissibility of acquired resistance, and survival time of individuals on treatment with acquired resistance, as well as the infectiousness of sensitive strains from treated cases, are sensitive factors of transmitted resistance prevalence [4, 27]. Acquired resistance prevalence is sensitive to the survival time of individuals on treatment with acquired resistance, treatment coverage, and treatment failure during the use of ART in the first year [27]. Thus, inadequate treatment should be avoided to reduce total drug-resistant HIV [58]. On the long run, the secondary transmission of drug resistance contributes significantly to its overall prevalence, which is related to the number of HIV infections in the pool [49, 57].

So far, no clinical trials provide data about drug resistance following the initiation of PrEP. There is a great need for the drug resistance on PrEP to be calibrated in a long run. There have been a considerable number of studies to date examining treatment resistance, but it is critical to calibrate the resistance to the results from models according to the dynamics of drug resistance in the real world. Although Supervie et al. [51] concluded the extent to which drug resistance is attributed to PrEP resistance or treatment resistance, it is too difficult for the differences between the two strategies to be distinguished in observational studies. These differences combined with other conditions will indicate whether a strategy of PrEP or ART is more effective [59]. It is inevitable that drug resistance prevalence will increase over time [6, 49, 50] even if new drugs are developed to fight against drug-resistant HIV. Regardless, public health experts should be cautious in observing the influence of drug resistance on HIV prevention and treatment and in attempting to avoid HIV resurgence in the near future. Perhaps in the end the prevalence of drug resistance will reach saturation; a 70% prevalence was given by Cambiano et al. with a conservative incidence rate per year compared to data from natural settings [49, 58]. A threshold of drug resistance prevalence is necessary to produce and evaluate whether treatment reduces HIV transmission or promotes the spread of HIV.

## 6. HIV Testing and Risk Behavior as Indirect Factors of Treatment Affecting New HIV Infections

The use of ART can save lives, prolong survival time, and prevent mother-to-child transmission [60, 61], all of which can encourage at-risk populations to be tested. The reduction of fear and stigma from HIV/AIDS also encourages HIV-infected individuals with an unknown HIV status to seek HIV testing [61]. Regular HIV testing helps in identifying HIV positive cases and then encourages patients to take the next step, for example, treatment or risk behavior change. So the terms “testing and treatment” are often linked together in studies. The increase in HIV testing frequencies or diagnosis rates can prevent more new HIV infections [21, 35, 36, 62]. As a precondition of ART, testing and treatment are indispensable to preventing new cases of HIV [50, 63]. This may be a form of synergy between HIV treatment and testing, especially in aggressive “test and treat” strategies [50, 64]. HIV screening and therapy targeted at high-risk populations is cost-effective in controlling the epidemic [36, 42, 62]. Periodic testing among high-risk and low-risk populations is more practical and cost-effective [62]. However, some overly optimistic testing scenarios might be unacceptable in real settings and may provide unfeasible outcomes of incident cases and a false timeline for HIV elimination [21].

Risk behavior change is related to changes in AIDS incidence and death rates partly as a result of treatment. First, the increase in risk behavior over time is attributed to the fact that patients in a high-risk population on ART survive and have a higher quality of life than before [13]. Second, cumulative risk behavior among surviving HIV patients is caused by a longer survival time partly attributed to effective treatment. Data from natural settings indicates an increase in risk behavior and incidence following the use of ART among men who have sex with men in Australia, France, and Netherlands [14, 65, 66], but perhaps these risk behavior increases may take more responsibility for incidence increases than we have observed. Risk behavior increases can give rise to more new HIV cases [35, 42, 66–68], so sexual disinhibition may offset the effectiveness of ART or even conceal its preventative benefit [35, 42, 66–68]. But combined efficacy-proven interventions can overcome the effects of risky behavior [33]. This is another paradox between population-level behavior disinhibition and HIV reduction as a result of ART use. Risk behavior distribution among a population impacts the effectiveness of treatment, and a homogeneous distribution (where the risk behavior distribution among populations is uniform) may be superior to the heterogeneous one (where distribution is uneven) [24]. The change in risk behavior may also produce brief redundancy among interventions, but synergistic relationships emerge between treatment and other intervention components with the waning of risk behavior change over time [69]. It is critical to address the risk behavior disinhibition to avoid resurgent HIV epidemics [35, 70].

## 7. How to Address Outcomes from the Above-Mentioned Mathematical Models

A mathematical model is a simplified realistic world [71] and also a thought experiment based on data of high quality [43, 72]. There is a gap between reality and the model. Simple models tend to deviate more from reality but are comparatively easy to analyze, whereas complex models are close to reality but relatively more difficult to analyze and interpret [71]. Delva et al.  [73] proposed nine principles of HIV mathematical models targeted at modelers and consumers, covering objectives, structure, parameters, presentation, and limitations. The nine principles in sum can comprehensively evaluate a model. Models simulate different outcomes based on different assumptions. Those outcomes which may reflect the real world come from assumptions and input data. The representative input data represents another arm to strengthen the applicability of the outputs [74]. It may not be feasible to reflect the real situation of an HIV epidemic using mathematical models. However, understanding the possible trends and effects of different interventions is helpful to the policy development process in HIV prevention and control. If based on real inputs, outcomes over the short term will have better projections of the real world than those over the long term because of less variation in interventions. A mathematical model is the optimal strategy to understand the long-term outcome of any intervention, and under certain conditions it may even be the only method [75]. Conclusions regarding whether a disease will be eliminated or eradicated should be based on real and practical input parameters. We should pursue optimistic outcomes as a goal and view conservative outcomes as a lesson or a warning.

An extreme example based on Granich et al.'s model structure and assumptions [21] raised great concern and controversy that HIV could be “eliminated” within 10 years in South Africa if universal testing and treatment were provided. However, the assumption that almost all cases will be identified and treated is untenable. Wagner et al. [48] simulated different conclusions on more realistic assumptions using the same model structure as Granich et al.'s [21]. ART is a potent tool for averting HIV transmission, but it alone cannot eliminate HIV [48]. This conclusion has extensive support [42, 68, 76]. Many studies [26–28, 35, 43, 50] considered different scenarios in comparison with a baseline scenario that evaluated real conditions, and they found a relationship between the strength of ART or other effective evidence-based interventions and HIV epidemics. However, it cannot be denied that some overly optimistic scenarios [21, 43, 76] based on unreachable hypotheses reached implausible outcomes. Accordingly, it may be meaningful that more concern should be put on the possible effects of interventions and potential prevalent trends. Pessimistic scenarios warn that one or more interventions which are unsuitable or unsustainable will lead to HIV spreading more quickly [35].

Another study compared and systematically analyzed the outcomes from 12 mathematical models on the basis of standardized treatment scenarios in South Africa [26]. It analyzed the differences between model outcomes that were unexplained by differences in model structure but explained by parameters such as treatment eligibility, efficacy, and the drop-out rate. This study first used the same standard of treatment to simulate outcomes in different models and reminded us that the results of mathematical models can be analyzed using traditional statistical methods such as meta-analysis. Perhaps further analysis will provide us with more evidence to explain the differences between outcomes from different models.

## 8. Model Complexity and Real-World Challenges

Whether the outcomes of a model fit with the real world depends on whether model structure and input parameters are close to those in real world. However, many challenges are presented for models and the real world to solve. Model structures also show a simplified version of the real world. The indispensable aspects of the models will determine if the structure is fitting [74], but deciding which aspects are crucial is difficult. Real data can also cause some confusion. On the one hand, advances in ART clearly demonstrate that new HIV infections can be prevented by suppressing viral loads. Adherence with ART reduces the average mortality rate in people living with HIV by one-half [12], reduces sexual transmission of HIV-1 in serodiscordant couples by 96% [15], and reduces the frequency of vertical transmission to 0 [77]. In addition, a series of studies on PrEP [78–81] provided further evidence to control the spread of HIV. But this success comes at a price: overshadowing historical behavior change efforts that have dramatically reduced HIV infections [8, 21]. Though treatment has become a new tool for prevention, “test and treat” methodologies must not undercut efforts in prevention that have always been, and remain, essential to curbing the epidemic [82].

Unfortunately, the CDC estimates that only 25% of the 1.1 million people living with HIV in USA in 2012 have achieved viral suppression (defined as ≤50 copies/mL) [83, 84]. The CDC considers ART with durable viral suppression key to a comprehensive HIV prevention strategy [85, 86]. In China, of the 780,000 people living with HIV, about 8% were estimated to have achieved viral load suppression in a pilot study (personal communication). The low rate reflects the challenges and gaps of HIV treatment as prevention, whether in the United States or China. Difficulties in retaining patients on ART and achieving viral suppression stem from delayed diagnosis and other challenges to engagement and retention in care, such as substance use, psychiatric disorders, language barriers, unstable housing or homelessness, incarceration, a busy work life, and side effects [87–90]. Even with improved access to ART through government initiatives, many patients struggle to take medications consistently. Regular adherence is crucial for long-term viral suppression, and missed doses or significant variation in dosage timing can lead to viral resistance that may portend treatment failure [91–93].

## 9. Conclusions

This review summarized the effectiveness of ART in preventing HIV transmission as simulated by dynamic mathematical models, and it is concerned with how to use ART among people with HIV for preventing HIV spreading at population level. As discussed in Section 4, HIV antiretroviral drugs as treatment alone cannot eliminate HIV epidemics, even under parameters whose values are feasible in the real world. Combined prevention methods given attention by policymakers can still play a larger role [28, 38, 69], especially in high-endemic resource-limited settings [25]. This analysis also revealed that there are synergistic effects among combinations of proven-efficacy interventions [69]. On the whole, there are still some controversies and problems to solve on the issue of “HIV treatment as prevention” [14, 16], and further modeling studies are needed.

Due to the latent infection, ART cannot cure HIV or eradicate it. HIV patients must take medicines continuously and tolerate the side effects throughout their lives. Currently new drugs such as histone deacetylase inhibitors, immunotherapy, and protein kinase C activators may control HIV replication and eventually eliminate HIV by activating the latent HIV reservoir [94, 95], but the effect must be further confirmed before these drugs are put on the market. Once drugs are able to cure HIV, the event will have a profound influence on HIV history. Models must adapt their model structures to simulate the current status of HIV transmission. New factors, such as cure and reinfection, will be incorporated appropriately in the model structure.
